# Exploring Personal Experience and Value Creation in Postdigital Education: Insights from a Large-Scale MOOC Survey

**DOI:** 10.1007/s42438-025-00561-0

**Published:** 2025-06-12

**Authors:** Ali Soleymani, Maarten De Laat, Marcus Specht

**Affiliations:** 1 Center for Education and Learning, EWI, EEMCS, TU Delft, Delft, the Netherlands; 2 Centre for Change and Complexity in Learning, University of South Australia, Adelaide, Australia; 3 Present Address: Department of Communication and Cognition, TSHD, Tilburg University, Tilburg, the Netherlands

**Keywords:** Value creation framework, Postdigital education, MOOCs, Online assessment

## Abstract

This study explores how participants of massive open online courses (MOOCs) perceive value creation within online learning environments. Drawing on the value creation framework (VCF), we developed and empirically validated a questionnaire, which was completed by 1227 learners enrolled in MOOCs offered by TU Delft. The aim was to provide deeper insight into participants’ experiences and the perceived impact of MOOCs on their personal and professional development. More specifically, this research explores the immediate, potential, applied, realized, and transformative value creation cycles. Our findings reveal significant insights into the multifaceted impacts of study behavior on learners’ perceptions. Participants reported benefits such as skill acquisition, professional development, and enhanced confidence while highlighting areas needing improvement, such as practical application opportunities and course relevance. This study highlights the importance of aligning MOOC content with learner needs and providing ongoing support to maximize the educational value that online courses can offer. These insights contribute to understanding educational value in the postdigital age, advocating for the development and support of MOOCs to foster continued personal and professional growth.

## Introduction

Massive open online courses (MOOCs) have emerged as an influential instrument for democratizing education and providing lifelong learning opportunities in the rapidly evolving landscape of postdigital education (Yuan and Powell 2013; de Freitas et al. 2015). Despite their global reach and potential, concerns remain regarding their effectiveness, equitable access, and whether they genuinely democratize education or primarily benefit already privileged learners (Hansen and Reich 2015; Littlejohn and Hood 2018). The rapid expansion of digital technologies has transformed educational practices and fostered environments where traditional boundaries between the digital and physical dissolve (Haleem et al. 2022). In this context, MOOCs present the integration of digital technologies into lifelong and networked learning, which aligns with the hybrid characteristics of the postdigital condition (Jandrić et al. 2018). Postdigital education moves beyond viewing technology as an enhancement or supplement to traditional practices. Instead, it embraces the multi-dimensionality of digital systems with social, cultural, and material dimensions of education (Knox 2023). As Jandrić et al. (2018: 895) argue, the postdigital is ‘messy, unpredictable, digital and analog, technological and non-technological’ and fundamentally challenges oversimplified views of educational technology. Considering this viewpoint, MOOCs can serve as platforms for knowledge dissemination and spaces where learners and educators navigate new socio-technical realities.

MOOCs are often described as transformative, providing unprecedented access to high-quality learning resources to a diverse global audience (Liyanagunawardena et al. 2013). However, the narrative of MOOCs as transformative tools is not without significant critique. For example, Kizilcec et al. (2013) found that learners from more privileged backgrounds, such as those with prior educational achievement, are more likely to complete MOOCs, which highlights the differences in course completion rates. Knox (2016) also critically examines the humanist assumptions underlying MOOCs and explores how these platforms may extend the existing educational inequalities. He argues that MOOCs often assume a universal learner and neglect the diverse socio-economic backgrounds of participants, which can lead to qualifying certain groups over others.

This study is situated within a critical discourse, acknowledging both the opportunities and limitations of MOOCs as postdigital learning spaces. Evaluating the educational experience of MOOC participants is not as straightforward as evaluating traditional educational programs, due to several factors such as the large and diverse population, varying levels of engagement, difficulty in capturing qualitative data, and the limited tools for such evaluation (Douglas et al. 2019; Lundqvist et al. 2020; Veletsianos et al. 2016). Most existing evaluation models in online learning rely heavily on quantitative data, such as completion rates, clickstream behavior, and satisfaction surveys, which often fail to capture the complex, relational, and contextual nature of learning. From a postdigital perspective, this narrow datafication of teaching quality obscures the deeper pedagogical processes, learner agency, and educational purposes that shape meaningful engagement (Fawns et al. 2021). As Fawns and colleagues argue, evaluation should move beyond reductionist metrics to embrace ecological approaches that account for non-datafied understandings, such as learner experiences, pedagogical design, and dialogic interactions. Therefore, many traditional educational frameworks and theories have limitations in evaluating diverse and complex learning environments like MOOCs due to their strong emphasis on quantitative data, which may overlook subjective learner experiences (Dingyloudi and Strijbos 2015) or not be able to collect and evaluate culturally sensitive information (Kennedy et al. 2022).

To understand the educational procedure and student experience in complex learning environments like MOOCs, we need a theoretical foundation that could help us first capture both quantitative and qualitative data and, second, context-specific educational experiences in various settings. Exploring the previous research, we have identified several papers that critique and utilize the VCF by Wenger-Trayner et al. (2011) for assessing MOOCs, demonstrating their potential advantage as flexible and qualitative evaluative tools over traditional frameworks (Dingyloudi and Strijbos 2015; Kennedy et al. 2022; Patel et al. 2019; Guldberg et al. 2019). Adopting Fawns’ (2023) perspective, postdigital education requires us to critically assess technological integration and the broader ethical, political, economic, and social justice implications of educational practices. In this context, using Wenger-Trayner et al. (2011) VCF helps explore how learners negotiate value within MOOCs’ spaces that, despite offering unprecedented access, also carry risks of exacerbating inequalities and marginalizing certain groups. The study contributes to ongoing discussions about inclusive, ethical, and social approaches to designing and implementing postdigital learning environments by exploring these value dynamics.

The VFC categorizes value into five cycles: immediate, potential, applied, realized, and transformative. Immediate value refers to the initial benefits participants derive from engaging in MOOCs, such as acquiring new knowledge and skills. Potential value encompasses the future benefits learners anticipate gaining from their MOOC experiences, including building their (professional) network, enhanced career prospects, and increased confidence. Applied value is observed when learners apply the knowledge gained from MOOCs in their professional and personal lives, leading to tangible improvements in job performance and new project initiatives. Realized value manifests as significant achievements in learners’ careers or personal development, such as job promotions and entrepreneurial successes. Finally, transformative value reflects more profound, long-term changes in learners’ perspectives and behaviors, fostering lifelong learning habits and a greater appreciation for online education (Wenger-Trayner et al. 2011).

Previous research has highlighted the growing significance of MOOCs in addressing global educational challenges. For instance, Patel et al. (2019) adapted the VCF to evaluate the impact of MOOC-based learning on trachoma elimination practices at the local level. Their findings underscored the importance of MOOCs in providing relevant knowledge and skills to health workers, which they could then apply in their communities to combat trachoma. Similarly, Gamage et al. (2016) explored the effectiveness of MOOCs, highlighting how the VCF could identify quality variations in course design and delivery, thus emphasizing its advantages in providing clear insights into learners’ experiences and the overall effectiveness of MOOCs.

However, understanding the full extent of MOOCs’ impact requires a comprehensive examination of the value they create for learners. This study explores the multifaceted dimensions of value creation in MOOCs, leveraging the VCF developed by Wenger-Trayner et al. (2011). These studies demonstrate that the VCF is a powerful tool for understanding and improving the learner experience in online education, especially in the context of lifelong learning and professional development. However, despite its usefulness, more research is needed to refine the framework further and explore its application across diverse educational settings to ensure its broader applicability and effectiveness. Also, our aim is to highlight MOOCs’ potential to foster value creation that is not merely individualistic but deeply embedded in the postdigital era’s collective, collaborative, and interconnected character.

TU Delft has established itself as a leader in online education, reaching over 3.6 million learners globally through a robust offering of 200 MOOCs, ten online academic courses, and more than 60 professional education courses. The university’s pedagogical model emphasizes flexibility, personalization, and a learner-centered approach guided by inclusiveness, interactivity, and innovation. These MOOCs cover strategic themes like Energy Transition, Quantum Technology, and Artificial Intelligence. They are designed to meet the needs of diverse global learners, providing a rich, interactive learning experience with significant community engagement. Through data-driven course design, TU Delft continuously enhances the quality and relevance of its MOOCs, ensuring they align with the latest educational research and technological advancements (Extension School n.d).

Building on insights into the values that MOOCs create for users, our study aims to investigate the value creation process in MOOCs offered by TU Delft. By surveying 1267 participants from TU Delft’s MOOCs, we seek to answer the following research questions:

1. What immediate value do participants derive from engaging in MOOCs, and how does this initial engagement influence their overall learning experience?
2. How do participants perceive the potential value of MOOCs in terms of social connection?
3. How do participants apply the knowledge gained from MOOCs in their professional and personal lives, and what factors influence this application?
4. What kind of realized values can be observed among MOOC participants and why?
5. To what extent do MOOCs facilitate transformative learning experiences that significantly change learners’ perspectives, practices, or worldviews, and what specific forms do these transformative experiences take?

By exploring the types of value articulated by MOOC participants through the VCF lens, this study aims to critically assess the multifaceted impacts of MOOCs on learners’ personal and professional development. Rather than presuming MOOCs deserve continued development and support, the study evaluates their perceived value to inform such decisions. Our results can offer insights into how learners experience value in online learning environments. These insights can contribute to the design of more inclusive and impactful MOOCs while also informing broader conversations about the role of digital education in the postdigital age, particularly concerning learner expectations, social justice, and access.

## Methodology

This study adopts a descriptive approach using the VCF by Wenger-Trayner et al. (2011), which categorizes value creation into five distinct cycles: immediate, potential, applied, realized, and transformative.

### Participants

We invited 35,000 previous TU Delft MOOCs’ participants via email. A total of 1960 participants responded to our invitation and completed our questionnaire. And 773 participants did not fill in the questionnaire completely. Therefore, we collected 1227 responses.

### Materials

Building upon Wenger-Trayner et al. (2011) five-cycle value creation model, the value creation questionnaire (VCQ) was developed to assess participants’ perceptions of value generated through their MOOCs at multiple levels. The questionnaire comprised ten items, equally distributed between closed-ended (multiple-choice) and open-ended questions. One multiple-choice and one open-ended query represented each of Wenger et al.’s value creation cycles.

For instance, participants were asked to rate their agreement (on a Likert scale of 1 (strongly disagree) to 6 (strongly agree)) with the statement, ‘Participation changed me as a student (change in skills, attitudes, identity, self-confidence, feelings, etc.).’ Positive responses (strongly agree, agree, or slightly agree) prompted an open-ended follow-up question: ‘Can you elaborate on how participation changed you as a student?’ Conversely, negative responses (slightly disagree, disagree, or strongly disagree) triggered a different open-ended question: ‘Can you explain why participation did not change you as a student?’ The full version of the questionnaire is attached as Appendix 1.

### Procedure

The study was conducted through an online survey distributed via email to 35,000 previous participants of TU Delft’s MOOCs. Participants were informed about the purpose of the study and provided consent before completing the survey. The survey was available for four weeks, and one reminder was sent to maximize the response rate.

### Qualitative and Quantitative Data Analysis

Data were analyzed using a mixed-methods approach. Quantitative data were analyzed using descriptive and inferential statistics with SPSS software. Thematic analysis was conducted on qualitative data using ChatGPT (OpenAI 2024) on 13 May 2024.

### LLM Use in Data Coding

#### Introduction of Researcher-Driven AI-assisted Qualitative Data Analysis (RDAQDA)

RDAQDA method (Nguyen-Trung 2024) was used to analyze the qualitative data from VCQ open-ended response. This method includes four stages of analysis using the LLMs. They are data familiarization, preliminary coding, template formation and refinement, and theme development. This method combines human expertise with AI capabilities to enhance the efficiency of our thematic analysis.

#### Detailed Analysis Process

Qualitative analysis involved the following steps:

- Step 1: Data familiarization. In this part, researchers thoroughly reviewed the dataset to gain an in-depth understanding of the content and context. Then, we tried to summarize and take note of the most important points mentioned in the answers. To empower our analysis using ChatGPT as an LLM, we asked ChatGPT to do the same task with the following prompt:
- Prompt 1. Can you please provide me with a summary of key ideas from all the responses in the uploaded file? [upload the first of the responses].

To make it manageable and compare the summary of ChatGPT with the researchers’ summary, we separated the responses into ten smaller parts. For example, responses about immediate values were separated into positive (part 1) and negative responses (part 2), positive responses to potential values question (part 3), and negative responses to potential value (part 4) based on the participant’s response to the first multiple-choice question. Therefore, in the end, we had ten separate parts. In the next steps, researchers compare their summaries with ChatGPT’s output and a final summary document for each of the parts created.

- Step 2. Preliminary coding. In this step, our goal was to create a list of initial codes for each of the parts that we explained in the data familiarization step. In this step, to create a context and make sure ChatGPT understands the goal of the research, the research questions included in our prompt. The following prompt was used to extract the initials codes for each of the subparts:
- Prompt 2. You’re a qualitative research assistant. You will help me identify the relevant codes from the following text in response to this question: ‘Question one about the immediate value?’ Our research question regarding this question is: ‘Research question one’. Codes are labels that assign summative, salient, essence-capturing, and/or evocative attributes/meanings to a portion of data. The final outputs are a table like this: Column 1: Code; Column 2: Description of code meaning; Column 3: Quotation representing the code from the responses. Here is the transcript: [insert the transcript].

The researcher reviewed the table of initial codes to make sure that each response was assigned to the correct code by ChatGPT.

- Step 3: Template refinement. In this step, the aim is to compact the initial codes and data to construct the clusters and then modify and finalize these initial categories. Therefore, the next prompt that has been used is as follows:
- Prompt 3. From the above refined list of codes, please group codes into clusters (i.e., more abstract codes) in response to the question: ‘Question one and two’. Remember that our research question was ‘Research question one’. For each of the clusters, please retain the specific codes and quotations from specific responses. If two or more responses fall under the same cluster, please put them together. The final output will be a table: Column 1: Cluster; Column 2: Codes; 3. Description of Cluster Meaning; Column 4: Quotation from responses.

Then, we followed the same prompt for each of the parts. Each of the created clusters was manually checked by the researcher to make sure that the responses clustered correctly.

- Step 4: Theme development. In this final step, the goal is to create four main themes for each of the parts based on the previously redefined clusters. Therefore, we used the following prompt to extract these themes:
- Prompt 4. From the above table of clusters, please generate themes across the clusters and codes in response to the question: ‘[questions 1 and 2 (about immediate value)]’ where we investigate this research question: ‘[research question 1 (about immediate value)]’. The theme is defined as the recurrent and distinctive features of participants’ accounts that characterize perceptions and/or experiences as you, as the researcher, see them as relevant to the research question of a particular study. Each theme should link the cluster(s) and/or code(s) with the context of ‘[immediate value]’. Themes must be relatively distinct from each other, although some overlap is inevitable. The final output will be a table: Column 1: Theme; Column 2: Clusters and Codes used for the theme; 3. Description of Theme Meaning; Column 4: Quotation from Transcripts.

By creating separated parts, we tried to maintain analytical consistency and reduce potential biases during AI-assisted analysis. Also, the coding template and themes were iteratively refined, by incorporating feedbacks and insights from both human analysts and AI outputs.

## Results

### Descriptive Analysis of VCQ

The descriptive analysis of the VCQ demonstrates participants’ experiences across the five value creation cycles: immediate, potential, applied, realized, and transformative. A full version of VCQ can be found in Appendix 1. Also, the results indicated in Table 1 provide insights into the average scores for each cycle, along with their standard deviations. As we explained in the methodology section, for each cycle, we have one multiple-choice question, and participants can respond on a Likert scale of 1–6.

**Table 1:** Descriptive analysis of VCQ

Value creation cycle	Average score	SD	Number of positive values	Percentage of positive values	Number of negative values	Percentage of negative values
Immediate	4.86	0.95	1146/1227*	93.40%	81/1227	6.60%
Potential	3.27	1.52	569/1227	46.43%	658/1227	53.63%
Applied	4.98	0.97	1156/1227	94.21%	40/1227	5.70%
Realized	4.11	1.40	870/1227	70.90%	357/1227	29.09%
Transformative	4.61	1.19	1060/1227	86.39%	167/1227	13.61%

*1227 is the total number of collected responses

As shown in Table 1, the applied value creation cycle received the highest average score of 4.98 (SD = 0.97), indicating that participants felt TU Delft MOOCs helped their practices. This finding was closely followed by the immediate value creation cycle results, with an average score of 4.86 (SD = 0.95), suggesting that participants obtained significant value from the initial engagement and activities within the MOOCs. The transformative cycle also scored high, with an average of 4.61 (SD = 1.19), reflecting how the courses led to more profound and meaningful changes in participants'perspectives and practices.

In contrast, the potential cycle, which relates to the effect of the course on participants’ social connection, had a lower average score of 3.27 (SD = 1.52), indicating variability in participants’ perceived potential value. The realized cycle, representing the long-term impact and effectiveness of the MOOCs, received a moderate average score of 4.11 (SD = 1.40), highlighting the challenges in sustaining the benefits of learning over time.

To better understand responses to multiple-choice questions, we divided them into positive and negative. It means that responses such as ‘strongly agree’, ‘agree’, and ‘slightly agree’ are considered positive, and ‘slightly disagree’, ‘disagree’, and ‘strongly disagree’ are considered negative. Table 1 summarizes this analysis of multiple-choice responses. Our analysis shows that only 569 out of all 1227 collected (46.43%) responses perceived the potential value positively. The highest positive responses belong to the applied cycle with 94.20% and the immediate value with 93.40%.

### Qualitative Analysis Open-End Responses

Participants were prompted with different follow-up open-ended questions based on the positive or negative responses to the multiple-choice questions. Thematic analysis of the open-ended responses from VCQ provides a better understanding of participants’ experiences, broken down into positive and negative responses to each cycle of values. By analyzing these responses, we can identify the most effective educational processes in the MOOCs, as well as areas where students face challenges. These two perspectives not only highlight the strengths of MOOCs in terms of immediate implementation and practical application but also highlight the need for improvement in areas where participants expressed challenges, particularly in terms of emphasizing the realization and social connection.

Thematic analysis of positive responses to the immediate value creation showed that participants experienced significant benefits, particularly knowledge transfer, skills development, confidence building, and career growth. A summary of these findings is shown in Table 2, and a full description of identified themes, their descriptions, and example quotes can be found in Tables 3, 4, 5, 6, and 7 in Appendix 2. Many students reported gaining new knowledge and insights in various areas. Other participants emphasized how it helped them gain new skills and increased their confidence in their abilities. For example, one participant mentioned that the course gave them confidence and knowledge that was previously lacking.

**Table 2:** Thematic analysis of open-ended response in VCQ

Value creation cycle	Positive/Negative	Theme
Immediate	Positive	Knowledge Enrichment
		Skill Development
		Confidence Boost
		Career Advancement
	Negative	No Practical Application
		Expectation Mismatch
		No Hope for Change in Their Career
		Course Difficulty
Potential	Positive	Networking and Social Connections
		Shared Learning Experiences
		Confidence and Personal Growth
		Global Engagement and Cultural Exchange
	Negative	Lack of Social Interaction
		Self-Paced and Individual Learning
		Limited Participation in Forums
		Online Format Constraints
Applied	Positive	Professional Development
		Learning and Education benefits
		Personal Growth
		Innovation and Inspiration
	Negative	No Impact on Professional Development
		No Personal Enrichment
		Lack of Relevance
		Professional Development
Realized	Positive	Solidified knowledge
		Boost in academic pursuit
		Enhanced self-confidence
		Positive Impact on professional development
	Negative	No Impact on Personal Development
		Lack of Practical Application
		Limited Impact on External Environment
		No Perceived Value and Recognition
Transformative	Positive	Insight On Environmental Consciousness
		Better understanding of Energy Transition
		Global Impact
		Problem-Solving Skills
	Negative	Enhanced Knowledge but no impact on worldview
		Improved Technical Skill Development but no impact on global perspective
		No General Impact on Worldview
		Insufficient Depth for Worldview Change

On the other hand, analysis of negative responses identified several areas of dissatisfaction among participants. Some felt the courses needed more practical opportunities with content or approaches that did not meet initial expectations. Some students found the courses challenging or too challenging. In contrast, others felt that the course had little or no impact on their career or personal development, highlighting the need for MOOCs to fill the gaps.

The thematic analysis of the open-ended responses about potential benefit cycles reveals positive and negative experiences regarding communication and social relationships among MOOC participants. On the positive side, about 46% of the respondents made new connections and expanded their networks. Some participants noted the value of shared learning experiences through group projects, which resulted in an improved sense of community.

In contrast, around 54% of participants expressed dissatisfaction with the social aspects of the courses. Many reported that they did not communicate with others, and they noted that the Internet limitation prevented them from communicating with others. Others mentioned that their learning style limited their social opportunities, and many did not participate in seminars or group activities. These findings suggest that while MOOCs can improve connections and bring value and cultural shifts, some online programs and these self-directed courses can prevent social interaction, too.

The third part of VCQ explores the applied value creation cycle, and participants are asked to respond to the following question: ‘Participation in TU Delft MOOC helped my practices as a student or a professional (get new ideas, insights, materials, procedures, etc.). For example, it helped you get a new idea of how you can work more efficiently or access materials you have not had before’. ‘Can you explain why participation did not help your practices as a student or a professional?’ Alternatively, ‘Can you explain how participation helped your practices as a student or a professional?’ Therefore, besides the first question, which is a multiple-choice question, we asked a follow-up open-ended question to let our participant elaborate on their first response.

TU Delft MOOCs created positive applied value for their participants. The vast majority of participants mentioned these courses’ impacts on their professional development. It means that the new knowledge and skills gained during the courses helped them to improve their professional role. This impact was not only in terms of professional development or advances in their career, but also in personal growth. In an interesting case, a participant mentioned that the online courses helped him balance work, life, and school.

On the other hand, a very small part of participants (around 4%) mentioned that these MOOCs had no impact on their professional development because they did not relate to the course materials or did not use them for academic or professional growth.

Evaluating the perceived realized value is challenging because this cycle of value is context-related. It takes time for professional learning network members to understand and realize this value in many situations. Nevertheless, around 70% of our participants answered positively to the following statement: ‘Participation in TU Delft MOOC changed my ability to influence my world as a student or a professional (enhance my voice, contribution, status, recognition, etc.)’. For example, it helped you to raise your voice and affect the organization that you are working for or the school that you are studying. The themes identified in open-ended responses include solidified knowledge, academic pursuit boost, self-confidence boost, and positive impact on professional development.

On the negative side, the remaining participants mentioned that these MOOCs had no practical influence on their career or study path. Participants were not able to influence their world/or surroundings with the knowledge and skills gained during the course.

Around 86% of participants claimed that TU Delft MOOCs made them see the world differently and explained that these courses helped them better understand different topics like environmental challenges, energy transition, and connected global challenges. Finally, one of the positive identified values was improving analytical and problem-solving skills, which helped the participants see their world differently. Besides these positive values, participants also mentioned that although these courses helped them gain new knowledge and skills, they did not necessarily change their views or perspectives about their surrounding worlds.

## Discussion

This study employed Wenger-Trayner et al. (2011) VCF to study the perceived values that TU Delft MOOCs offer. Our findings highlight positive outcomes and the areas needing improvement, offering valuable insights for educators, policymakers, and researchers.

### Immediate Value

To answer our first research question, which was the immediate value participants derived from engaging in MOOCs, our results show that 93.40% of our participants perceived the positive immediate value of participating in MOOCs. They described these positive values as gaining new knowledge, developing new skills, feeling more confident, and seeing the hope for career advancement. On the other hand, 6.60% of participants expressed concern about mismatched expectations, lack of hope, frustration with a career change after doing the courses, and course difficulty. This suggests a need for MOOCs to differentiate their content and delivery methods to ensure that they offer unique and valuable learning experiences (Ucha 2023).

The central identified positive theme was the confidence boost. Our participants described improved self-efficacy and confidence, like navigating technology and managing their time. These findings align with previous research on the impact of MOOCs on learners’ self-efficacy and confidence (Beirne et al. 2023, 2021).

Mismatched expectations can lead to decreased satisfaction and reduced intention to continue using MOOCs (Lee et al. 2024; Daneji et al. 2019). Although a small portion of our participants report this negative feeling while doing the MOOCs, it is one of the main negative themes identified in our study.

### Potential Value

Our second research question is about how our participants perceive the potential value of MOOCs in terms of social connection. Our finding reveals a mixed perception of this social connection in MOOCs as an indicator of potential value. Around 53% claimed these MOOCs had no positive impact on their social connections. Previous studies show that this social interaction can be mediated by immersive experience and psychological needs satisfaction (Fang et al. 2019). The identified theme in our studies shows that participants who emphasize their individual learning goals and have difficulty in online connections tend to interact less with their peers and be active on MOOC discussion forums.

However, this lack of social presence and interaction can increase the chance of drop-off and negative educational experiences (Zou et al. 2021; Estrada-Molina and Fuentes-Cancell 2021). Therefore, course instructors and designers must develop innovative ways to implement and improve the social presence and interaction in their MOOCs. For example, recently, researchers have tried to implement AI tools in MOOC to reach this goal (for review, Loh et al. 2024).

### Applied Value

In the VCF, values are described as interconnected cycles (Wenger-Trayner et al. 2011). Also, our analysis revealed an interesting correlation or trend between immediate, potential, and applied value. Thematic analysis of applied value shows learners were able to improve their professional development by using the knowledge and skills (immediate value) and connection (potential value) gained through the MOOCs.

To elaborate, participants highlighted improvements in job performance and implementing new ideas and practices in their everyday tasks. These outcomes demonstrate the practical benefits of MOOCs, enabling learners to apply theoretical knowledge to real-world scenarios, thus enhancing their professional capabilities. The flexible nature of MOOCs allows learners to balance their studies with professional commitments. This flexibility enables continuous learning and skill development, which are crucial for career advancement (Bralić and Divjak 2018).

However, the lack of practical application opportunities was a common criticism. Participants expressed the need for more hands-on activities and practical exercises to better translate theoretical knowledge into practical skills. This is especially true when we want to target working professionals in the MOOCs (Liu et al. 2020). Addressing this gap could involve integrating more interactive components like simulations, case studies, and project-based learning.

### Realized Value

Realized values are context-related and usually difficult to capture due to their long-term effect (Wenger-Trayner et al. 2011). This can explain why we observed around 70% positive value perception (in comparison with around 95% positive responses in immediate and applied value). Nevertheless, participants reported significant achievements and improvements in job performance due to their participation in MOOCs. These main positive realized values were the boost in academic pursuit, which included gaining knowledge about time-saving techniques, enhanced productivity, and positive impacts on academic performance metrics.

On the other hand, some participants did not perceive significant improvements in their performance, and the central theme revealed was a limited impact on the external environment. This points to the necessity of providing ongoing support and follow-up resources to help learners effectively implement what they have learned (Rotar 2022).

### Transformative Value

The positive transformative impact of MOOCs was evident in 86.39% of participants. They claimed that the MOOCs provided them with a deeper insight into the global impacts of environmental challenges and fundamental topics like energy transition. Environmental challenges and energy transition were two of the main themes of TU Delft MOOCs. Moreover, the results show that these two themes successfully changed the learners’ perspectives and strategies when approaching these global concerns. Also, other research on the impact of MOOCs on global challenges shows that universities and their MOOCs can significantly contribute to knowledge exchange and professional networks’ knowledge development (Laurillard and Kennedy 2020).

However, not all participants realized the transformative potential. Some indicated that the courses needed to provide more depth or relevance to prompt a shift in their worldview. To explain this lack of perceived value, research has shown that a lack of teaching-based quality and insufficient depth in study materials in MOOCs can significantly decrease learning efficiency (Abhishek et al. 2023; Shanshan and Wenfei 2022). This suggests a need for MOOCs to offer more in-depth content and opportunities for critical reflection to foster transformative learning experiences.

### Implications for Practice

One of our goals in this research is to explore and evaluate MOOCs through learners’ eyes. VCQ enables us to explore learners’ behavior, perceived value, engagement, and satisfaction using qualitative and quantitative measures. Therefore, investigating MOOCs from the learner’s perspective can help utilize learner-centered design that supports the needs and intentions of their participants embedded in the postdigital society (Moore and Blackmon 2022).

Our finding that learners perceive significant value in skill acquisition aligns with previous MOOC research emphasizing the importance of practical skills development for learner motivation (Lee and Song 2022). Our finding also resonates with broader research on online learning that highlights the link between perceived relevance and learner engagement (Li et al. 2023; Pan 2023). Furthermore, this finding supports the principles of authentic learning design, which advocate for learning experiences that are connected to real-world contexts and applications (e.g., Lombardi 2007; O'Neill and Short 2023).

Based on our findings in this research, we suggest considering the following features in designing a learner-centered MOOC.

#### Setting a Clear Course Expectation

Course designers need to clearly outline the course content, structure, and learning objectives of the MOOC and set up difficulty levels to manage learner expectations and reduce mismatch. This feature also enables learners to match their current knowledge and skills with the MOOC’s difficulty level. It increases learners’ overall enjoyable experience at the first stage of their learning programs. Also, the other recent finding confirms that addressing learner expectations and communicating the usefulness of the MOOC ensure learners’ satisfaction and continuance of the course (Rekha et al. 2023).

#### Fostering Networking Opportunities

By integrating collaborative projects, discussion forums, and an effective peer feedback system, we can implement networking opportunities in MOOCs. Encouraging learners to share their insights can create a valuable chance for them to build professional networks in the future (Soleymani et al. 2022). Also, MOOC designers need to keep in mind that just implementing discussion forums is not enough to have an effective social learning environment. The lack of support for social interaction (Rivera et al. 2024) and providing no quick feedback on contributions to the discussion forums (Wei et al. 2023) can negatively influence the student experience in MOOCs.

#### Encouraging Professional Development via Real-World Cases and Relevant Content

Course content significantly predicts MOOC retention via perceived effectiveness (Hone and El Said 2016). Therefore, our results show that when our participants did not understand the relevance of the course content to their field, and there were no real-world case studies in the MOOC, they were less likely to use or apply what they had learned in their everyday practice as students or professionals. The MOOC can be designed to support the transfer of online learning into offline action (Napier et al. 2020).

#### Implementing the ‘Impact Reflection Assignments’

One solution to raise awareness about the positive realized value among our learners is encouraging them to think beyond their learning environment. Motivate them to consider how they can influence their surroundings (e.g., their organization or school). This can be done by what we call an ‘Impact Reflection Assignment’. This assignment includes reflective activities where learners assess and discuss how their new knowledge and skills can influence and improve their external environment. Previous findings also confirm the fundamental role of reflection facilitation in improving the quality of the learning experience for learners (Daalhuizen and Schoormans 2018).

#### Big Picture Design

Many TU Delft-provided MOOCs consider a bigger picture or theme while designing these educational programs. These big pictures are, for example, related to the environment, sustainability, energy transition, or AI in society. We believe this was a successful strategy. Our results here show that most of our participants, after completing the MOOCs, start to think beyond their boundaries and consider the global impacts of different topics like energy and environment. Course designers can implement in-depth exploration content or encourage critical debates in their MOOCs to promote positive, transformative value.

### Using Natural Language Processing (NLP) Tools for Thematic Analysis

ChatGPT is one of the most widely used NLP tools, with a growing number of applications in different fields, especially education research (Albadarin et al. 2024). A growing number of researchers use such tools for their thematic analysis to identify and interpret patterns in research data (Lee et al. 2024). ChatGPT is a valuable tool for research, especially for thematic analysis, as it enhances efficiency and provides additional insight into the qualitative data. However, there are essential challenges when using ChatGPT for thematic analysis. These challenges include hallucination of the large language model (LLM) (for example, when the produced responses by LLM are not justified by the data used to feed the model), privacy issues, and high prompt dependency (for example, requesting the same output in the prompt with different phrased can generate different output) (De Paoli 2024). Therefore, when using NLP tools like ChatGPT, we need to consider the critical role of interaction between the human researcher and the tool to ensure reliable results.

### Limitations

While this study offers valuable insight into learners’ perception of value creation in MOOCs, several limitations must be acknowledged. First, one of the main concerns is the applicability of our results. This study surveyed participants from MOOCs offered exclusively by TU Delft, which may limit the generalizability of the findings to other institutions or contexts. Also, learners in these MOOCs may differ in demographic or professional backgrounds compared with other MOOCs offered by different organizations, which can potentially affect the applicability of our results to the broader population.

Second, although our qualitative analysis using ChatGPT facilitated an efficient thematic analysis, it also has some limitations. The quality of the AI-assisted coding depended heavily on the clarity of prompts and the researchers’ validation of AI outputs. Despite our careful and manual checking of the process, some responses may have been overlooked.

Third, while the VCF provides a structured lens for our analysis, it is important to acknowledge the inherent subjectivity in interpreting and measuring perceived value. Besides this, we did not include the time interval between the MOOCs’ end and the time participants answered our value creation questionnaire. Therefore, the timeframe and subjective nature of value creation might affect the results.

Finally, this research relies heavily on the qualitative results of our survey. While this approach allows for the analysis of large datasets like MOOCs and the identification of general trends, it provides limited insight into the complexities of individual learning experiences. Qualitative research methods, such as interviews or focus groups, could complement these findings by providing richer and in-depth data.

### Implications for Theory

This study contributes to theoretical discussions of learning design, value creation, and postdigital education by critically examining how learners perceive and experience value in MOOCs. Drawing on Wenger-Trayner et al. (2011) VCF, we extended its application to large-scale, asynchronous, and highly heterogeneous learning environment contexts traditionally considered difficult to evaluate meaningfully through qualitative or learner-centered models. Our findings demonstrate that learners articulate value not only in terms of immediate knowledge gain or skill acquisition but also through relational, emotional, and identity-related transformations, which align with calls in postdigital theory to move beyond reductive, data-driven models of educational effectiveness (Fawns et al. 2021).

This work supports and deepens the notion that postdigital education involves more than delivering content through digital platforms. Instead, it is embedded in complex social, technological, material, and ethical contexts (Fawns 2023). Our findings challenge dominant evaluation paradigms that often focus on completion rates or behavioral data and offer an alternative theoretical lens that captures learners’ multi-layered, value-driven experiences in networked environments. By showing how MOOCs can foster applied and realized learning and transformative shifts in worldview, we contribute to a richer conceptualization of what “learning” means in postdigital spaces.

Furthermore, this study invites theoretical reconsideration of how value creation is distributed, perceived, and potentially constrained by structural inequalities. While MOOCs are frequently positioned as democratizing forces, our findings reveal a more complex picture: value is not equally accessible or experienced. This echoes critiques in postdigital literature that emphasize the socioeconomic and political dimensions of education and the need for inclusive, socially just learning design (Jandrić et al. 2024; Fawns 2023).

By integrating a value-based, learner-centered theoretical framework with postdigital education’s critical, ecological sensibilities, this study contributes to ongoing efforts to theorize learning not merely as acquisition but as situated practice shaped by individual, institutional, and sociotechnical relations. It reinforces the call for educational research to adopt frameworks sensitive to both learners’ lived experiences and the broader systemic forces at play in postdigital educational ecosystems.

## Conclusion

This study provides a comprehensive analysis of the value creation process in MOOCs offered by TU Delft, revealing significant and multifaceted impacts on learners. The insights gained highlight the strengths and areas for improvement in MOOC design and delivery, contributing to a better understanding of the role of digital education in fostering personal and professional growth. By addressing the identified gaps and leveraging the potential of MOOCs, educators and institutions can enhance the learning experiences and outcomes for a diverse global audience.

## Data Availability

The data that support the findings of this study are available from the Dutch Research Council (NWO), but restrictions apply to the availability of these data, which were used under license for the current study and so are not publicly available. The data are, however, available from the authors upon reasonable request and with the permission of the Dutch Research Council (NWO).

## Appendix 1

### Value Creation Questionnaire

1. Participation in TU Delft MOOC changed me as a student or professional (change in skills, attitudes, identity, self-confidence, feelings, etc. [for example, it helped you to gain a new skill]).

1. A. Can you explain why participation did not change you?

1. B. 2 B. Can you explain how participation changed you?

2. Participation in TU Delft MOOC affected my social connections (change in the number, quality, frequency, emotions, etc. [for example, it helped you find x new number of connections or friends]).

2. A. Can you explain why participation didn't affect your social connections?

2. B. Can you explain how participation affected your social connections?

3. Participation in TU Delft MOOC helped my practices as a student or a professional (get new ideas, insights, materials, procedures, etc. [for example, it helped you to get a new idea on how you can work more efficiently, or got access to materials that you haven’t had before]).

3. A. Can you explain why participation didn't help your practices as a student or a professional?

3. B. Can you explain how participation helped your practices as a student or a professional?

4. Participation in TU Delft MOOC changed my ability to influence my world as a student or a professional (enhance my voice, contribution, status, recognition, etc.[for example, it helped you to raise your voice and affect the organization that you are working for or the school that you are studying]).

4. A. Can you explain why participation didn't change your ability to influence your world as a student or a professional?

4. B. Can you explain how participation changed your ability to influence your world as a student or a professional?

5. Participation in TU Delft MOOC made me see my world differently (change in perspective, new understandings of the situation, redefine success, etc. [for example, it helped you redefine how to see a problem and gave a new approach to solve it]).

5. A. Can you explain why participation didn't make you see your world differently?

5. B. Can you explain how participation made you see your world differently?

## Appendix 2

Table 3

Table 4

Table 5

Table 6

Table 7

**Table 3:** Thematic analysis of VCQ open-ended responses—immediate value

Positive/negative	Theme	Description	Quote example
Positive	Knowledge Enrichment	Participants gained new knowledge and insights in various fields.	It broadened my knowledge on water purification methods and gave me insight in how calculations such as for pressure within Reverse Osmosis worked.
	Skill Development	Participants reported acquiring new skills and enhancing existing ones.	It helped me gain a new skill and knowledge.
	Confidence Boost	Engagement in courses increased participant’s confidence in their abilities and knowledge.	It gave me the confidence and knowledge that I lacked on the subject.
	Career Advancement	Participants felt the courses assisted them in their career growth and development.	Participation in the Coursework helped me acquire a lot of knowledge in the field of biomedical engineering.
Negative	No Practical Application	Participants felt the course lacked practical knowledge and application opportunities.	Didn’t get enough practical knowledge or the chance to exercise what I learned, while working in the construction industry.
	Expectation Mismatch	Participants had expectations about course content and format that were not met.	I expected a much different course. There were very few video lectures and notes.
	No Hope for Change in Their Career	Participants felt the course had little impact on their career or personal development.	It didn’t have a big impact on my career or personal development.
	Course Difficulty	Participants found the course challenging or too complex.	The first module was way too complicated for me.

**Table 4:** Thematic analysis of open-ended responses—potential value

Positive/negative	Theme	Description	Quote example
Positive	Networking and Social Connections	Participants mentioned forming new connections and expanding their networks.	Met a friend and we are still very much in contact. Increased my network connections.
	Shared Learning Experiences	Engagement in group projects led to shared learning experiences and connections with diverse peers.	Collaborating on group projects created a sense of community.
	Confidence and Personal Growth	Participants reported increased confidence levels and personal growth through interactions in the course.	Boosted my confidence to participate in discussions.
	Global Engagement and Cultural Exchange	Engaging with diverse participants led to global engagement and cultural exchange.	Exposure to diverse perspectives enhanced my learning.
Negative	Lack of Social Interaction	Participants mentioned the absence of social opportunities and interaction in the online courses.	Because I never talked to anyone from Delft or within the edX Pre-University Calculus community.
	Self-Paced and Individual Learning	Participants focused on individual learning goals without actively seeking social connections.	I took the course at my own pace and did not communicate with anyone else.
	Limited Participation in Forums	Many participants did not actively engage in the course forums or discussions.	Not much social interaction and did not participate in group activities.
	Online Format Constraints	Challenges related to the online format restricting social interactions among participants.	Being an online course, it was not manageable to maintain any connections.

**Table 5:** Thematic analysis of open-ended responses—applied value

Positive/negative	Theme	Description	Quote example
Positive	Professional Development	Participants benefited in their professional roles, gaining new knowledge and skills.	Gave me insight into EVs and also some of the people/companies working in that space.
	Learning and Education benefits	Participants highlighted the value of learning and education from the courses.	The abundance of study materials and lecture videos have given me a very good learning experience and understanding complex topics in a detailed and fun way.
	Personal Growth	Participants experienced personal growth and development through the courses.	To plan my time properly between work, personal life and school.
	Innovation and Inspiration	Participants mentioned gaining new ideas, perspectives, and inspiration from the courses.	TU Delft MOOCs often cover cutting-edge topics and research findings in various fields. By participating in these courses, you expose yourself to new ideas and perspectives that can inspire innovation and creativity in your academic or professional endeavors. Whether it’s learning about emerging technologies, novel theories, or innovative solutions to real-world problems, these insights can broaden your thinking and challenge conventional wisdom.
Negative	No Impact on Professional Development	Participants did not find direct impact on their professional practices or studies.	Participation didn’t directly impact my practices as a student or professional because I haven’t actively engaged with the resources provided by the TU Delft MOOC. While I acknowledge the potential benefits, I have yet to explore and utilize them effectively to enhance my practices. Work in software development. Not AI related (YET).
	No Personal Enrichment	Participants took courses for personal interest and enrichment without professional or academic gain.	For me the courses were purely personal and had no professional or practical purpose. They were mostly taken for personal enrichment, I am out of the workforce and not likely to return to school at this point, so there was no professional nor academic gain to be had.
	Lack of Relevance	Participants highlighted that the courses did not cover new techniques, study tips, or were not relevant to their studies or professional life.	Because a lot of it didn’t apply to my studies. It was just informational. It wasn’t relevant for my studies.
	Limited Engagement	Participants mentioned limited engagement, lack of practical use, or no impact due to factors like unfamiliar subject areas.	No influence. No ideas have occurred to me yet. I didn’t engage enough for it to have an impact.

**Table 6:** Thematic analysis of open-ended response in VCQ—realized value

Positive/negative	Theme	Description	Quote example
Positive	Solidified knowledge	Enhanced knowledge and understanding gained from the course.	I can apply the knowledge in my profession.
	Boost in academic pursuit	Participants experienced a confidence boost and presence enhancement in their academic journey.	The participation built my presence which gave me a boost in my academic pursuit.
	Enhanced self-confidence	Increased self-confidence and ability to communicate with others.	I feel more confident to talk with researchers or professional around the world.
	Positive Impact on professional development	Improved skills, knowledge, and abilities relevant to professional growth and challenges.	As a professional I can push my boundaries to take up new challenges in combination of power and process.
Negative	No Impact on Personal Development	Participants’ personal growth and development through the course.	Although I gained insight it had no effect as I struggle to find community within that niche in South Africa. The course had more of a personal effect than an effect on my professional career.
	Lack of Practical Application	Absence of practical skills gained from the course.	Only theory ideas, no developed practical competencies. New skills were not directly applicable to my work.
	Limited Impact on External Environment	Participants expressing minimal influence on their surrounding world.	It changed ME of course but I couldn’t change anything outside of me. I don’t think I have changed anything around me.
	No Perceived Value and Recognition	No change on views on the perceived value and recognition of MOOC participation.	With no expendable recognition, MOOC falls into the category of personal interest/hobby. For some individuals … it could very easily act as a career enabler.

**Table 7:** Thematic analysis of open-ended response in VCQ—transformative value

Positive/negative	Theme	Description	Quote example
Positive	Insight On Environmental Consciousness	Recognizing the role of sustainable practices.	I have come to recognize the critical role of electric vehicles (EVs) in reducing greenhouse gas emissions. Understanding EV technology connects me to the broader energy transition.
	Better understanding of Energy Transition	Understanding the shift towards sustainable energy sources.	My awareness extends beyond personal benefits. I realize that every EV on the road contributes to a collective effort toward a healthier planet. As I delve into EV infrastructure and charging solutions, I have come to appreciate the impact on urban planning.
	Global Impact	Acknowledging the interconnectedness of global challenges.	It helped me gain insights into the field and the technology associated with it. The course helped me create a more in-depth knowledge on the indicated topic.
	Problem-Solving Skills	Enhancing critical thinking and analytical abilities.	Engaging with the material and software has provided me with a deeper appreciation for the complexities of hydrodynamic processes. The course taught me the function and exactly how aircraft can fly.
Negative	Enhanced Knowledge but no impact on worldview	Participants felt that the course added to their existing knowledge without fundamentally changing their worldview.	It just enhanced my knowledge a little. But that’s not enough to say that I see the world differently. Although I learned a lot, it was more for a better understanding rather than new insights that changed my views.
	Improved Technical Skill Development but no impact on global perspective	Focus on acquiring technical skills rather than altering perspective on the world.	Although I learned a new skill, it was not in an area that changed my worldview. I took a technical course and hence it gave me a way to look at technical problems related to that domain differently, but it didn’t affect the rest of my decisions.
	No General Impact on Worldview	Participants expressed that the course did not have a significant impact on how they see the world.	No, I’m convinced my view of the world is quite good. Participation in the TU Delft MOOC has not brought about any changes in how I see my world. It has not changed my perspective.
	Insufficient Depth for Worldview Change	Feedback indicates that the course content lacked depth or relevance to prompt a shift in worldview.	Although it was mind-boggling, I was kind of known to the facts provided. The content of the course was in line with expectations. It gave more depth that I had before, but it was not earth-shattering.

## References

[CR1] Abhishek, A., Kulal, A., M.S., D., & Dinesh, S. (2023). Effectiveness of MOOCs on learning efficiency of students: A perception study. *Journal of Research in Innovative Teaching & Learning. *10.1108/JRIT-12-2022-0091.

[CR2] Albadarin, Y., Saqr, M., Pope, N., & Tukiainen, M. (2024). A systematic literature review of empirical research on ChatGPT in education. *Discover Education, 3*(1), 60. 10.1007/s44217-024-00138-2.

[CR3] Beirne, E., Nic Giolla Mhichíl, M., Brown, M., & Mac Lochlainn, C. (2021). Confidence counts: Fostering online learning self-efficacy with a MOOC. In *Proceedings of EMOOCs 2021* (pp. 201–208). 10.25932/publishup-51722.

[CR4] Beirne, E., Nic Giolla Mhichíl, M., Brown, M., & Mac Lochlainn, C. (2023). Clicking with confidence: Influence of a student co-designed MOOC on students’ emotions and online learning self-efficacy. *Online Learning, 27*(2). 10.24059/olj.v27i2.3758.

[CR5] Bralić, A., & Divjak, B. (2018). Integrating MOOCs in traditionally taught courses: Achieving learning outcomes with blended learning. *International Journal of Educational Technology in Higher Education, 15*(1), 2. 10.1186/s41239-017-0085-7.

[CR6] Chan, H. P., & King, I. (2017). Leveraging social connections to improve peer assessment in MOOCs. In *Proceedings of the 26th international conference on world wide web companion* (pp. 341–349). New York: Association for Computing Machinery. 10.1145/3041021.3054165.

[CR7] Daalhuizen, J., & Schoormans, J. (2018). Pioneering online design teaching in a MOOC format: Tools for facilitating experiential learning. *International Journal of Design, 12*(2), 1–14.

[CR8] Daneji, A. A., Ayub, A. F. M., & Khambari, M. N. M. (2019). The effects of perceived usefulness, confirmation and satisfaction on continuance intention in using massive open online course (MOOC). *Knowledge Management & E-Learning: An International Journal, 11(*2).

[CR9] De Paoli S (2024) Performing an inductive thematic analysis of semi-structured interviews with a large language model: An exploration and provocation on the limits of the approach. *Qualitative Health Research 34*(3), 521–532. 10.1177/08944393231220483.

[CR10] Dingyloudi, F., & Strijbos, J. (2015). Examining value creation in a community of learning practice: Methodological reflections on story-telling and story-reading. *Seminar Net, 11*(3). 10.7577/seminar.2348.

[CR11] Douglas, K., Zielinski, M., Merzdorf, H., Diefes-Dux, H., & Bermel, P. (2019). Meaningful learner information for MOOC instructors examined through a contextualized evaluation framework. *International Review of Research in Open and Distance Learning, 20*(1), 205–220. 10.19173/irrodl.v20i1.3717.

[CR12] Estrada Molina, O., Fuentes Cancell, D. (2021). Engagement and desertion in MOOCs: Systematic review. *Comunicar 30*(69), 9–20. 10.3916/C69-2021-01.

[CR13] Extension School. (n.d.). TU Delft. https://www.tudelft.nl/extension-school. Accessed 17 Mar 2025.

[CR14] Fang, J., Tang, L., Yang, J., & Peng, M. (2019). Social interaction in MOOCs: The mediating effects of immersive experience and psychological needs satisfaction. *Telematics and Informatics, 39*, 75–91. 10.1016/j.tele.2019.01.006.

[CR15] Fawns, T. (2023). Postdigital education. In P. Jandrić (Ed.), *Encyclopedia of postdigital science and education.* Cham: Springer. 10.1007/978-3-031-35469-4_52-1.

[CR16] Fawns, T., Aitken, G., & Jones, D. (2021). Ecological teaching evaluation vs the datafication of quality: Understanding education with, and around, data. *Postdigital Science and Education, 3*(1), 65–82. 10.1007/s42438-020-00109-4.

[CR17] Freitas, S., Morgan, J., & Gibson, D. (2015). Will MOOCs transform learning and teaching in higher education? Engagement and course retention in online learning provision. *British Journal of Educational Technology, 46*(3), 455–471. 10.1111/bjet.12268.

[CR18] Gamage, D., Perera, I., & Fernando, S. (2016). Evaluating effectiveness of MOOCs using empirical tools: Learners perspective. In *Proceedings of INTED2016* (pp. 1183–1192). 10.21125/inted.2016.0937.

[CR19] Guldberg, K., Achtypi, A., D’Alonzo, L., Laskaridou, K., Milton, D., Molteni, P., & Wood, R. (2019). Using the value creation framework to capture knowledge co-creation and pathways to impact in a transnational community of practice in autism education. *International Journal of Research & Method in Education, 44*(1), 1–16. 10.1080/1743727X.2019.1706466.

[CR20] Haleem, A., Javaid, M., Qadri, M. A., & Suman, R. (2022). Understanding the role of digital technologies in education: A review. *Sustainable Operations and Computers, 3*, 275–285. 10.1016/j.susoc.2022.05.004.

[CR21] Hansen, J. D., & Reich, J. (2015). Democratizing education? Examining access and usage patterns in massive open online courses. *Science, 350*(6265), 1245–1248. 10.1126/science.aab3782. 26785488 10.1126/science.aab3782

[CR22] Hone, K. S., & El Said, G. R. (2016). Exploring the factors affecting MOOC retention: A survey study. *Computers & Education, 98*, 157–168. 10.1016/j.compedu.2016.03.016.

[CR23] Jandrić, P., Knox, J., Besley, T., Ryberg, T., Suoranta, J., & Hayes, S. (2018). Postdigital science and education. *Educational Philosophy and Theory, 50*(10), 893–899. 10.1080/00131857.2018.1454000.

[CR24] Jandrić, P., MacKenzie, A., & Knox, J. (2024). Postdigital research: Genealogies, challenges, and future perspectives. *Postdigital Science and Education, 6*(2), 409–415. 10.1007/s42438-022-00306-3. 10.1007/s42438-022-00306-3 PMC8959268 40479248

[CR25] Kennedy, E., Masuda, C., Moussaoui, R. E., Chase, E., & Laurillard, D. (2022). Creating value from co-designing CoMOOCs with teachers in challenging environments. *London Review of Education, 20*(1). 10.14324/LRE.20.1.45.

[CR26] Kizilcec, R. F., Piech, C., & Schneider, E. (2013). Deconstructing disengagement: Analyzing learner subpopulations in massive open online courses. In *LAK '13: Proceedings of the third international conference on learning analytics and knowledge *(pp. 170–179). New York: Association for Computing Machinery. 10.1145/2460296.2460330.

[CR27] Knox, J. (2016). *Posthumanism and the massive open online course: Contaminating the subject of global education*. New York: Routledge. 10.4324/9781315674032.

[CR28] Knox, J. (2023). Educational development in the postdigital era. In W. O. Lee, P. Brown, A. L. Goodwin, & A. Green (Eds.), *International handbook on education development in Asia-Pacific.* Singapore: Springer. 10.1007/978-981-16-2327-1_119-1.

[CR29] Laurillard, D., & Kennedy, E. (2020). The role of higher education in upscaling global professional development through open, online collaboration. London: UCL Knowledge Lab, Institute of Education.

[CR30] Lee, Y., & Song, H.-D. (2022). Motivation for MOOC learning persistence: An expectancy–value theory perspective. *Frontiers in Psychology, 13*. 10.3389/fpsyg.2022.958945. 10.3389/fpsyg.2022.958945 PMC9426658 36051216

[CR31] Lee, V. V., Van Der Lubbe, S. C. C., Goh, L. H., & Valderas, J. M. (2024). Harnessing ChatGPT for thematic analysis: Are we ready? *Journal of Medical Internet Research, 26, *e54974*.* 10.2196/54974. 10.2196/54974 PMC11179012 38819896

[CR32] Li, L., Zhang, R., & Piper, A. M. (2023). Predictors of student engagement and perceived learning in emergency online education amidst COVID-19: A community of inquiry perspective. *Computers in Human Behavior Reports, 12*. 10.1016/j.chbr.2023.100326.

[CR33] Littlejohn, A., Hood, N. (2018). The [Un]democratisation of education and learning. In: Reconceptualising learning in the digital age. SpringerBriefs in Education. Springer, Singapore. 10.1007/978-981-10-8893-3_2.

[CR34] Liu, M., Zou, W., Shi, Y., Pan, Z., & Li, C. (2020). What do participants think of today’s MOOCs: An updated look at the benefits and challenges of MOOCs designed for working professionals. *Journal of Computing in Higher Education, 32*(2), 307–329. 10.1007/s12528-019-09234-x.

[CR35] Liyanagunawardena, T. R., Adams, A. A., & Williams, S. A. (2013). MOOCs: A systematic study of the published literature 2008–2012. *The International Review of Research in Open and Distributed Learning, 14*(3), 202–227. 10.19173/irrodl.v14i3.1455.

[CR36] Loh, H. S., Martins van Jaarsveld, G., Mesutoglu, C., & Baars, M. (2024). Supporting social interactions to improve MOOC participants’ learning outcomes: A literature review. *Frontiers in Education, 9*. 10.3389/feduc.2024.1345205.

[CR37] Lombardi, M. (2007). Authentic learning for the 21st century: An overview. EDUCAUSE Learning Initiative. https://library.educause.edu/resources/2007/1/authentic-learning-for-the-21st-century-an-overview. Accessed 29 May 2025.

[CR38] Lundqvist, K., Liyanagunawardena, T., & Starkey, L. (2020). Evaluation of student feedback within a MOOC using sentiment analysis and target groups. *The International Review of Research in Open and Distributed Learning, 21*(3), 133–151. 10.19173/irrodl.v21i3.4783.

[CR39] Moore, R. L., & Blackmon, S. J. (2022). From the learner’s perspective: A systematic review of MOOC learner experiences (2008–2021). *Computers & Education, 190*. 10.1016/j.compedu.2022.104596.

[CR40] Napier, A., Huttner-Loan, E., & Reich, J. (2020). Evaluating learning transfer from MOOCs to workplaces: A case study from teacher education and launching innovation in schools. *RIED Revista Iberoamericana De Educación a Distancia, 23*(2), 45. 10.5944/ried.23.2.26377.

[CR41] Nguyen-Trung, K. (2024). ChatGPT in thematic analysis: Can AI become a research assistant in qualitative research? OSF Preprints. 10.31219/osf.io/vefwc.

[CR42] O’Neill, G., & Short, A. (2023). Relevant, practical and connected to the real world: What higher education students say engages them in the curriculum. *Irish Educational Studies, 42*(4), 717–734. 10.1080/03323315.2023.2221663.

[CR43] Pan, X. (2023). Online learning environments, learners’ empowerment, and learning behavioral engagement: The mediating role of learning motivation. *Sage Open, 13*(4). 10.1177/21582440231205098.

[CR44] Patel, D., Leck, A., McCormick, I., Kennedy, E., & Parsley, S. (2019). Value creation framework to assess MOOC-based learning. In *Proceedings of PCF Scotland 2019*. British Columbia: Commonwealth of Learning (COL). http://hdl.handle.net/11599/3259. Accessed 29 May 2025.

[CR45] Rekha, I. S., Shetty, J., & Basri, S. (2023). Students’ continuance intention to use MOOCs: Empirical evidence from India. *Education and Information Technologies, 28*(4), 4265–4286. 10.1007/s10639-022-11308-w. 36259079 10.1007/s10639-022-11308-w PMC9561332

[CR46] Rivera, D. A., Frenay, M., & Swaen, V. (2024). The learning design of MOOC Discussion Forums: An analysis of forum instructions and their role in supporting the social construction of knowledge. *Technology Knowledge and Learning, 29*(2), 585–615. 10.1007/s10758-023-09670-w.

[CR47] Rotar, O. (2022). Online student support: A framework for embedding support interventions into the online learning cycle. *Research and Practice in Technology Enhanced Learning, 17*(1), 2. 10.1186/s41039-021-00178-4.

[CR48] Shanshan, S., & Wenfei, L. (2022). Understanding the impact of quality elements on MOOCs continuance intention. *Education and Information Technologies, 27*(8), 10949–10976. 10.1007/s10639-022-11063-y. 35498957 10.1007/s10639-022-11063-y PMC9039272

[CR49] Soleymani, A., Itard, L., Laat, M. de, Torre, M. V., & Specht, M. (2022). Using social network analysis to explore learning networks in MOOCs discussion forums. *Proceedings of CLIMA 2022*. 10.34641/clima.2022.300.

[CR50] Ucha, C. R. (2023). Role of course relevance and course content quality in MOOCs acceptance and use. *Computers and Education Open, 5*. 10.1016/j.caeo.2023.100147.

[CR51] Veletsianos, G., Reich, J., & Pasquini, L. A. (2016). The life between big data log events: Learners’ strategies to overcome challenges in MOOCs. *AERA Open, 2*(3). 10.1177/2332858416657002.

[CR52] Wei, W., Liu, J., Xu, X., Kolletar-Zhu, K., & Zhang, Y. (2023). Effective interactive engagement strategies for MOOC forum discussion: A self-efficacy perspective. *PLOS ONE, 18*(11). 10.1371/journal.pone.0293668. 10.1371/journal.pone.0293668 PMC10635535 37943846

[CR53] Wenger-Trayner, E., Trayner, B., & Laat, M. (2011). Promoting and assessing value creation in communities and networks: A conceptual framework. Ruud de Moor Centrum. http://bsili.3csn.org/files/2013/06/Wenger_Trayner_DeLaat_Value_creation.pdf. Accessed 29 May 2025.

[CR54] Yuan, L., & Powell, S. (2013). MOOCs and open education: Implications for higher education. Center for educational technology & interoperability standards. 10.13140/2.1.5072.8320.

[CR55] Zou, W., Hu, X., Pan, Z., Li, C., Cai, Y., & Liu, M. (2021). Exploring the relationship between social presence and learners’ prestige in MOOC discussion forums using automated content analysis and social network analysis. *Computers in Human Behavior, 115*. 10.1016/j.chb.2020.106582.

